# Poling-free integrated second-order nonlinear optics with evaporated organic thin films

**DOI:** 10.1126/sciadv.aeg3170

**Published:** 2026-05-27

**Authors:** Pierre-Luc Thériault, Arnaud Petit, Abhay Anand V.S., Stéphane Kéna-Cohen

**Affiliations:** Department of Engineering Physics, Polytechnique Montréal, Montréal, H3T 1J4, Canada.

## Abstract

Integrated second-order [χ^(2)^] photonics underpins next-generation classical and quantum technologies, enabling applications ranging from frequency conversion and high-speed modulation to entangled photon generation. However, the field is now limited by a lack of materials that combine high nonlinear performance with scalable, CMOS-compatible fabrication. Vapor-deposited organic thin films having large χ^(2)^ nonlinearities without electric field poling offer a versatile solution, theoretically enabling both standalone organic circuits and heterogeneous integration on passive platforms. Yet, the translation of these films into functional photonic devices has remained speculative. Here, we demonstrate phase-matched second-harmonic generation in strip-loaded waveguides by harnessing the film’s giant birefringence (Δ*n* ≈ −0.2) to match fundamental modes (TE_00_ → TM_00_), thereby maintaining high modal overlap. The resulting efficiency of $\eta_{L}=29\%W^{-1}{\text{cm}}^{-2}$ rivals that of strip-loaded waveguides on thin-film lithium niobate. These results establish spontaneously oriented organics as a promising material class for the integration of second-order nonlinear functionalities on arbitrary substrates.

## Teaser

Poling-free organic films offer a scalable route to efficient second-order nonlinear optical processes on photonic chips.

## INTRODUCTION

The rapid expansion of integrated silicon photonics has revolutionized on-chip data processing (*1*) by enabling high-bandwidth optical interconnects (*2*), high-capacity optical transceivers (*3*), and scalable neural network accelerators (*4*, *5*). Yet, the platform remains fundamentally limited by the centrosymmetry of silicon, which precludes intrinsic second-order nonlinear [χ^(2)^] effects. Integrated χ^(2)^ nonlinear processes are key enablers for next-generation photonic circuits, allowing for on-chip light sources (*6*), entangled pair generation (*7*), and efficient high-speed modulation (*8*). To overcome this limitation, the field has pursued two primary strategies: the development of new monolithic platforms that offer intrinsic χ^(2)^ functionality, such as thin-film lithium niobate (TFLN) (*9*), or the heterogeneous integration of noncentrosymmetric materials onto silicon or silicon nitride (*10*, *11*). While TFLN now sets the performance standard for monolithic platforms, its high-temperature processing and potential for lithium-ion contamination hinder monolithic complementary metal-oxide semiconductor (CMOS) integration (*12*). Conversely, heterogeneous integration provides a versatile route to add χ^(2)^ functionality to passive centrosymmetric platforms, but it is constrained by material compatibility and process complexity (*13*).

Organic materials offer a compelling alternative to this heterointegration challenge, as they can be deposited onto virtually any CMOS-compatible platform using simple, low-temperature processing (*14*, *15*). By leveraging the enormous nonlinearities of organic molecules (*14*), this approach has the potential to enable high-performance nonlinear and electro-optic devices without the complex bonding or epitaxial growth required for inorganic crystals. However, a fundamental challenge remains: Because conventional solution-processed films are centrosymmetric, harnessing their potential typically requires electric field poling to break this symmetry. This step involves applying a high static electric field across the film to force the naturally randomized molecular dipoles into alignment. This technique has proven highly effective, facilitating the development of ultrafast electro-optic modulators that have now reached commercial deployment (*16*). Nevertheless, the poling requirement adds substantial fabrication complexity and necessitates the use of metal electrodes (*17*). For purely all-optical processes like frequency conversion or optical parametric oscillation, these electrodes introduce unnecessary optical loss (*18*) and increase device footprint without providing any functionality.

**Fig. 1.**
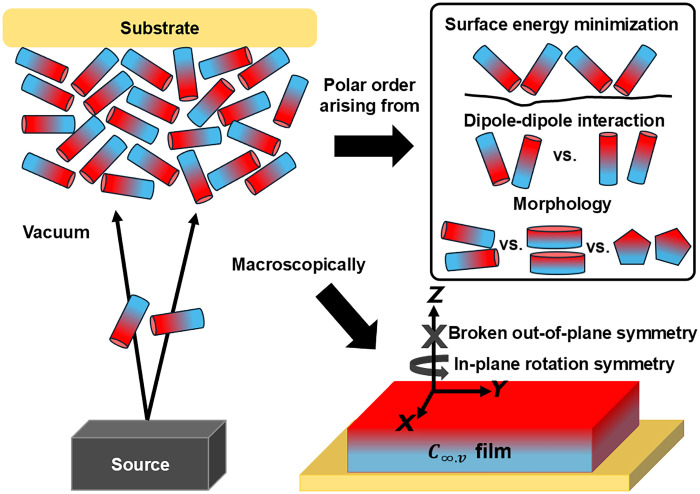
Conceptual illustration of spontaneous orientation in vapor-deposited organic thin films. Molecules sublimate from a source in vacuum and self-assemble upon condensation on an arbitrary substrate. Macroscopically, this process results in an amorphous film with *C*_∞,*v*_ symmetry, which has broken out-of-plane symmetry (along the *Z* axis) but in-plane rotational symmetry. This net polar order arises from a delicate balance of competing intermolecular forces and surface effects, including surface energy minimization, dipole-dipole interactions, and preferred molecular packing (morphology).

We recently identified several nonlinear compounds that can spontaneously break symmetry during vapor deposition, thus avoiding the need for poling altogether (*19*). In this case, the noncentrosymmetric *C*_∞*v*_ structure emerges naturally during deposition, driven by surface energy minimization and competing intermolecular forces (Fig. 1) (*20*–*22*). This spontaneous orientation translates the large microscopic nonlinearities of the constituent molecules into large macroscopic χ^(2)^ nonlinearities. Beyond their large nonlinearity [up to 43 pm/V at λ = 1.27 μm and 18 pm/V at λ = 1.55 μm (*23*)], these films often have a large uniaxial birefringence, providing additional dispersion engineering flexibility to achieve the phase-matching conditions required for efficient nonlinear interactions (*24*).

**Fig. 2.**
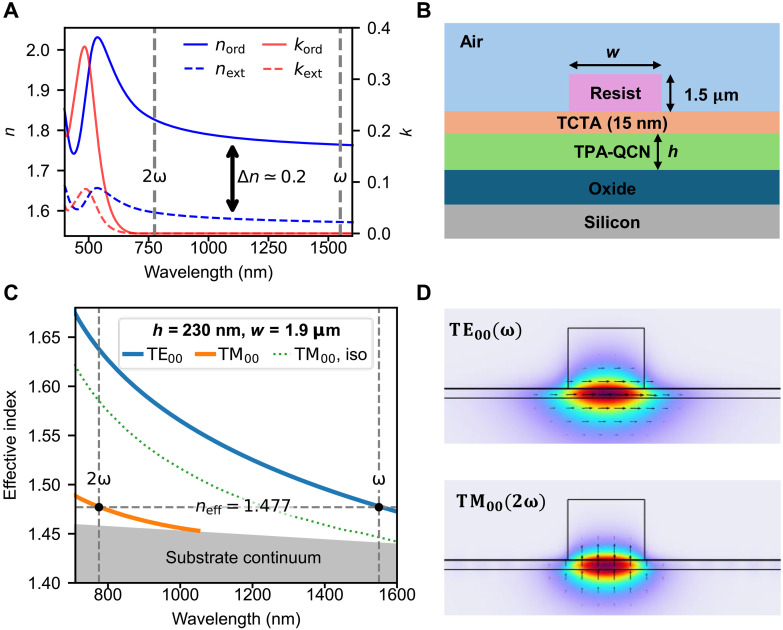
Birefringent phase-matching design and simulation. (**A**) Measured optical constants (*n* and *k*) of the spontaneously oriented TPA-QCN film. The plot reveals a giant, negative uniaxial birefringence (Δ*n* = *n*_ext_ − *n*_ord_ ≈ −0.2). (**B**) Schematic of the etchless strip-loaded waveguide. (**C**) Simulated effective index dispersion for the target geometry (*h* = 230 nm, *w* = 1.9 μm). The giant birefringence from (A) enables the TE_00_ (ω) mode (blue curve) to be phase-matched with the fundamental TM_00_ (2ω) mode (orange curve) at *n*_eff_ = 1.477. The dotted green line (TM_00,iso_) shows the nonphase-matched isotropic case, highlighting the necessity of the birefringence. (**D**) Simulated mode profiles for the phase-matched TE_00_ (ω) and TM_00_ (2ω) modes.

This unique combination of a large poling-free χ^(2)^, high intrinsic birefringence, and the potential for heterointegration on arbitrary substrates is highly promising, but component-level implementations have remained speculative. Here, we demonstrate second-harmonic generation (SHG) in strip-loaded waveguides using vapor-deposited films. By exploiting the large birefringence (Δ*n* = *n*_ext_ − *n*_ord_ ≈ −0.2 where *n*_ext_ and *n*_ord_ are respectively the extraordinary and ordinary refractive indices; Fig. 2A), we achieve phase matching between two fundamental modes [TE_00_(ω) → TM_00_(2ω)], thereby maximizing the spatial overlap essential for high efficiency. This yields a length-normalized conversion efficiency of $\eta_{L}=29\%W^{-1}{\text{cm}}^{-2}$ without the need for electric field poling or periodic patterning, a value that compares favorably to state-of-the-art strip-loaded TFLN demonstrations (*25*, *26*). Notably, our analysis shows that avoidable radiation leakage now limits the efficiency, pointing to substantial room for improvement.

## RESULTS

### Principle of birefringent phase matching

The efficiency of SHG in a waveguide is governed by three key parameters: the material nonlinearity, the phase matching condition, and modal overlap within the nonlinear medium (*27*). While nonlinearity is an intrinsic material property, achieving phase matching is the primary engineering challenge for χ^(2)^ processes due to the large chromatic dispersion between octave-separated wavelengths. In poling-free platforms, the standard approach is modal phase matching, where the fundamental mode is matched to a higher-order mode at the second harmonic frequency. This strategy leverages modal dispersion to counteract chromatic dispersion, ensuring that the mode effective refractive indices (*n*_eff_) at frequencies ω and 2ω are equal [$n_{\text{eff}}\left(\omega \right)=n_{\text{eff}}\left(2\omega \right)$]. However, this strategy comes at a cost: The spatial profile of the higher-order mode typically overlaps poorly with the fundamental pump mode, inherently penalizing the conversion efficiency.

To overcome this difficulty, we use spontaneously oriented films of 2-(4′-diphenylaminobiphenyl-4-yl)quinoxaline-6,7-dicarbonitrile (TPA-QCN) (*19*, *28*). Its nonlinear susceptibility tensor yields two independent coefficients, $\chi_{33}^{\left(2\right)}=7\pm 3$ pm/V and $\chi_{31}^{\left(2\right)}=8.1\pm 0.3$ pm/V at 1550 nm. In waveguides, its large $\chi_{31}^{\left(2\right)}$ coefficient, 30% larger than its counterpart in LiNbO_3_ (*29*), enables coupling between a transverse electric (TE) polarized fundamental mode and a transverse magnetic (TM) polarized second-harmonic mode. Although normal material dispersion typically precludes phase matching between the fundamental modes ($n_{\text{eff},2\omega }>n_{\text{eff},\omega }$), the material’s large negative birefringence (Δ*n* ≈ −0.2; Fig. 2) counteracts this effect. Specifically, the negative birefringence lowers the effective index (*n*_eff_) of the TM_00_ mode at 2ω (sensitive primarily to the lower-valued extraordinary index), allowing it to phase-match with the TE_00_ mode at ω (sensitive only to the higher-valued ordinary index). While birefringent phase matching has also been explored in lithium niobate (LiNbO_3_) waveguides (*30*), compensating for normal dispersion with the material’s modest birefringence necessitates impractical waveguide dimensions. This renders the approach incompatible with dense integration, whereas our film’s large birefringence enables the process in compact, wavelength-scale structures.

### Device design and simulation

With spontaneously oriented thin films, birefringent phase matching can be applied to various waveguide geometries, including fully etched strip waveguides (Supplementary Text). To minimize fabrication complexity, we opted for an etchless, strip-loaded structure (Fig. 2B). The device consists of a 230-nm TPA-QCN active layer deposited on a silicon substrate with a 2-μm thermal oxide buffer. This thickness for the active layer was chosen to enable fundamental-mode phase matching (Fig. 2C and fig. S1) and single-mode operation at λ = 1550 nm while respecting the practical constraints of the fabrication process. A 15-nm 4,4′,4-tris(carbazol-9-yl)triphenylamine (TCTA) layer was subsequently deposited, serving as a protective layer and enhancing the TPA-QCN’s thermal stability (*31*). Last, a strip of a low-index resist (Fig. 2B) provides lateral confinement. The resist has a refractive index (fig. S2B) of *n* = 1.418 at λ = 1550 nm and *n* = 1.423 at λ = 775 nm, with no measurable absorption at either wavelength (within our instrument sensitivity).

In this configuration, phase matching is achieved by tuning the strip width (*w*). Simulations (Fig. 2C) show that this geometric tuning allows the ${\text{TE}}_{00}\left(\omega \right)$ mode to be perfectly phase-matched with the ${\text{TM}}_{00}\left(2\omega \right)$ mode for a strip width of *w* = 1.9 μm at 1550 nm. Crucially, because this interaction involves two fundamental modes with similar spatial profiles (Fig. 2D), the calculated spatial overlap integral is high [96%, following the treatment in (*27*)], which is a distinct advantage over modal phase-matching schemes. The critical role of the material’s intrinsic birefringence is illustrated by the dotted line, which simulates the ${\text{TM}}_{00}\left(2\omega \right)$ dispersion obtained in the isotropic case ($n_{\text{iso}}=n_{\text{ord}}$). In this hypothetical case, the index of the TM_10_ mode remains far too high, and no crossing occurs at 2ω. It is only the large negative birefringence that lowers the TM_00_(2ω) effective index sufficiently to enable fundamental-mode matching.

Furthermore, this design ensures high modal selectivity. While the waveguide supports other modes at 2ω (e.g., TE_10_ and TM_10_), conversion into these channels is forbidden. Conversion to TE modes is disallowed by the *C*_∞*v*_ tensor symmetry [$\chi_{11}^{\left(2\right)}=\chi_{22}^{\left(2\right)}=0$], and conversion to the TM_10_ mode is forbidden by the zero overlap integral between an even pump (TE_00_) and an odd harmonic (TM_10_) modes. Thus, all converted power is expected to be channeled into the desired TM_00_ mode, thereby maximizing conversion efficiency.

### Experimental demonstration

**Fig. 3.**
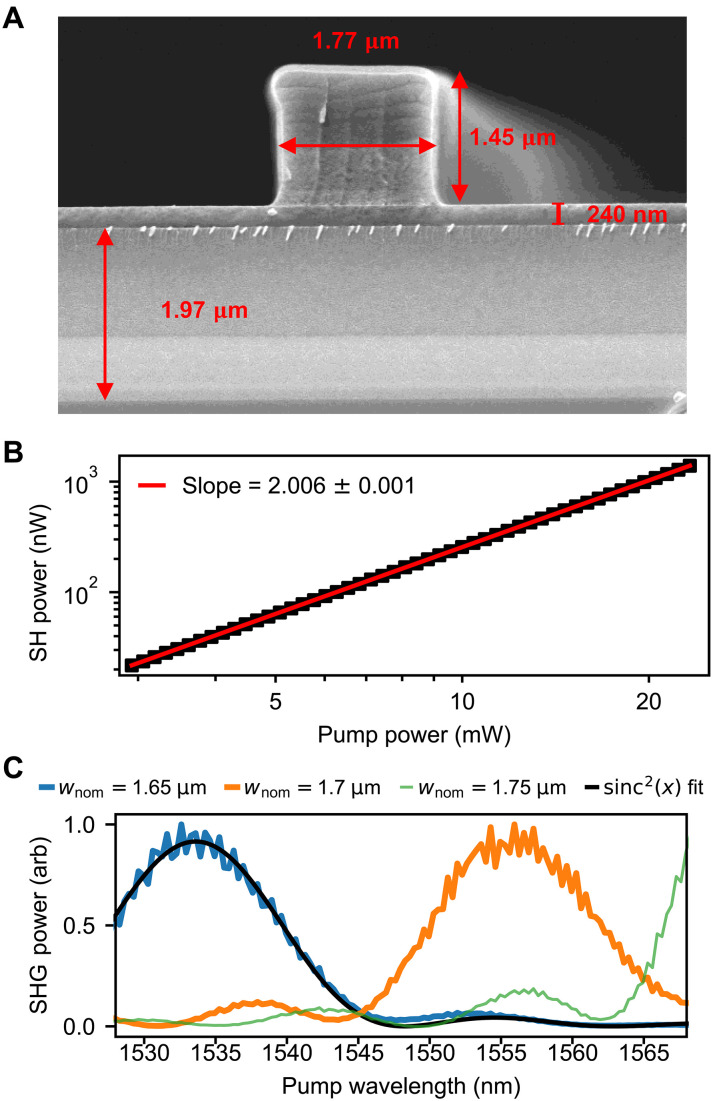
Experimental demonstration and characterization of phase-matched SHG. (**A**) SEM cross section of a fabricated strip-loaded waveguide. (**B**) Log-log plot of the SH power at the end facet versus the on-chip fundamental power at the input facet. The red line is a linear fit with a slope of 2.006 ± 0.001, confirming the quadratic nature of the χ^(2)^ process. (**C**) Normalized SHG power in arbitrary (arb) units as a function of fundamental wavelength for waveguides with different nominal strip widths (*w*_nom_). The spectra show prominent sinc^2^-like peaks (black fit line) that are geometrically tunable, providing a definitive signature of birefringent phase matching.

Devices were fabricated (Materials and Methods) with the simulated geometry shown in Fig. 2B. A scanning electron microscope (SEM) image of a typical waveguide cross section is shown in Fig. 3A. The resulting structure closely matches the desired structure, showing nearly vertical sidewalls. To characterize the nonlinear performance, we coupled a continuous-wave C-band tunable laser into the waveguides and measured the second-harmonic intensity from the output facet using a calibrated photodetector (Materials and Methods). The χ^(2)^ origin of the process can be confirmed by measuring the second-harmonic power dependence versus the input fundamental power. As shown in Fig. 3B, the resulting log-log plot yields a slope of 2.006 ± 0.001, in nearly perfect agreement with the expected quadratic relationship.

To explore the role of birefringent phase matching, we measured the pump wavelength dependence of the second-harmonic intensity (Fig. 3C) for waveguides with different nominal widths. For each waveguide, we observed a prominent central peak with distinct side lobes, consistent with the characteristic sinc^2^-like shape expected from a phase-matching process (*24*). Following the trend predicted by simulations (Supplementary Text), the peak phase-matching wavelength is geometrically tunable: Increasing the nominal strip width by ∼50 nm shifts the central peak by ∼22 nm. This demonstrates that the phase-matching condition is being controlled by the strip geometry, as designed.

### Loss analysis and efficiency

**Fig. 4.**
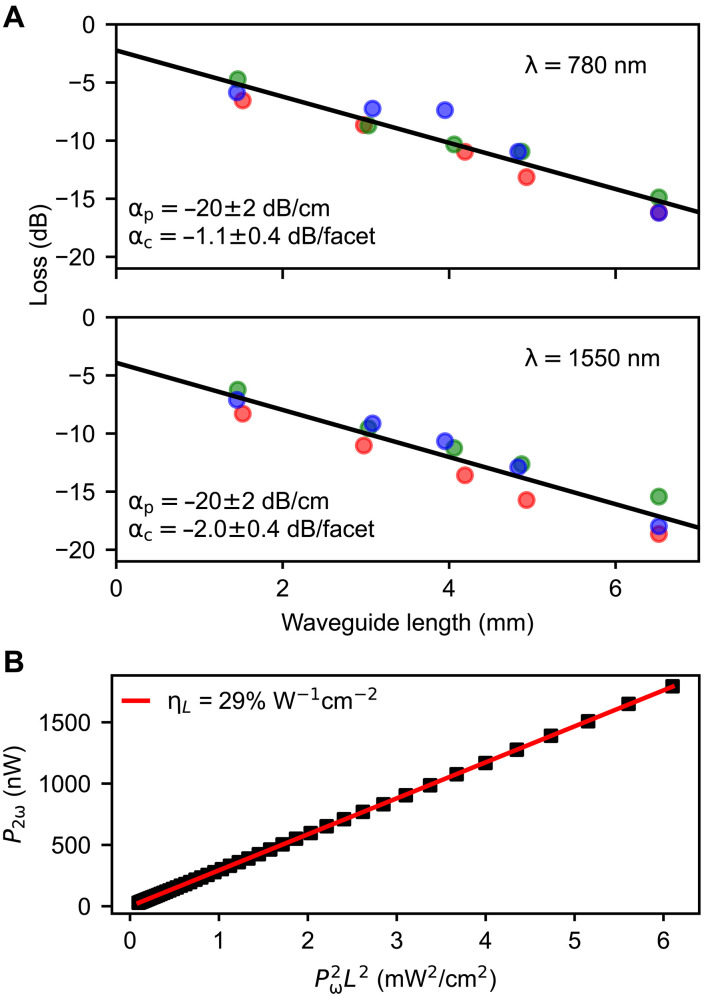
Propagation loss and conversion efficiency characterization. (**A**) Cut-back propagation loss measurements for different waveguides (colored markers) at the SH wavelength (λ = 780 nm, top) and the fundamental wavelength (λ = 1550 nm, bottom). The linear fit (black line) yields a propagation loss α_p_ = −20 ± 2 dB/cm and facet coupling losses α_c_ of −1.1 ± 0.4 dB per facet and −2.0 ± 0.4 dB per facet for the SH and fundamental, respectively. (**B**) Measured SHG power (*P*_2ω_) as a function of $P_{\omega }^{2}L^{2}$ for the best-performing 1.7-mm-long device, using the on-chip fundamental power (*P*_ω_) corrected for coupling loss. The linear fit (red line) gives a length-normalized conversion efficiency of η*_L_* = 29% W^−1^ cm^−2^.

To accurately quantify the conversion efficiency, we first characterized the optical losses. Propagation losses (α_p_) were measured via the cut-back method (Fig. 4A) at both the fundamental (ω ≈ 1550 nm) and second-harmonic (2ω ≈ 780 nm) wavelengths. Measurements revealed losses of α_p_ ≈ 20 ± 2 dB/cm for both bands. From the *y* intercept of the cut-back measurements, we also determined the coupling losses (α_c_) to be 2.0 ± 0.4 dB per facet at the fundamental and 1.1 ± 0.4 dB per facet at the SH wavelength. These efficient coupling values are attributed to the high facet quality achieved via cleaving, combined with the material’s low refractive index and large mode volume.

Using the corrected on-chip power *P*_ω_ (accounting for coupling losses), we calculated the length-normalized conversion efficiency $\eta_{L}=P_{2\omega }/\left(P_{\omega }^{2}L^{2}\right)$. For the best-performing 1.7-mm-long waveguide, we obtained a normalized efficiency of $\eta_{L}=29\%W^{-1}{\text{cm}}^{-2}$.

## DISCUSSION

We have demonstrated a functional, poling-free χ^(2)^ waveguide that serves as a critical step toward the use of spontaneously oriented organic thin films in integrated χ^(2)^ nonlinear photonics. By harnessing the giant intrinsic birefringence of spontaneously oriented TPA-QCN films, we achieved phase-matched SHG in simple strip-loaded waveguides without the need for periodic patterning or electric field poling. The resulting efficiency of $\eta_{L}=29\%W^{-1}{\text{cm}}^{-2}$ validates the potential of spontaneously oriented films for high-performance applications. It exceeds the efficiency of similar strip-loaded hybrid polymer-on-TFLN devices [$\eta_{L}\approx 0.2\%W^{-1}{\text{cm}}^{-2}$ (*25*)] by more than two orders of magnitude and approaches the performance of silicon nitride-loaded TFLN waveguides (*26*). While this initial demonstration does not yet rival mature, periodically poled monolithic TFLN [$\eta_{L}\approx 5000\%W^{-1}{\text{cm}}^{-2}$ (*32*)], it establishes a robust baseline for a new class of χ^(2)^ materials.

This demonstration also bridges a critical gap between material discovery and practical device implementation. Previous efforts to exploit organic χ^(2)^ materials have faced persistent trade-offs: Early work relied on one-dimensional (1D)–confined slab waveguides (*33*–*36*) incompatible with the dense routing required by modern integrated photonics, while 2D-confined devices have necessitated complex electric field poling (*37*–*41*). The latter introduces substantial fabrication complexity and requires electrodes that induce optical loss. While alternative poling-free techniques—such as lithography on single-crystal films (*42*) or direct growth of crystalline waveguides (*43*–*49*)—have been explored, they remain constrained by scalability issues and have yet to yield functional, efficient frequency conversion devices. By overcoming these limitations with a scalable, dry physical vapor deposition process, our approach provides a practical route to high-efficiency χ^(2)^ organic waveguides that are fully compatible with backend CMOS processing.

Crucially, our analysis indicates that the current performance is limited by addressable design constraints. At the SH wavelength, the intrinsic material absorption (*k* < 10^−6^) predicts propagation losses below 1 dB/cm, and the film is fully transparent at 1550 nm (*23*). Instead, simulations identify two dominant, easily avoidable loss channels that account for more than 10 dB/cm at both wavelengths: leakage into the silicon substrate through the thin 2-μm buffer and lateral leakage of the TM-polarized second-harmonic mode into TE slab modes (*50*). Both mechanisms can be suppressed through design improvements, such as increasing the buffer thickness and optimizing the strip dimensions to eliminate lateral leakage (Supplementary Text). Eliminating these leakage pathways would reduce losses to the current scattering level (5 dB/cm), which our calculations show would immediately boost the conversion efficiency by a factor of 2.3 to 68% W^−1^ cm^−2^ (Supplementary Text). Last, further process optimization is expected to significantly reduce these scattering losses.

Furthermore, the current performance could be improved through material optimization. The use of organic molecules allows for continuous property optimization and tuning. Recent work on TPA-QCN derivatives (*23*) has shown a twofold enhancement in second-order susceptibility compared to TPA-QCN, offering a potential additional fourfold increase in efficiency. Combining these design and material improvements points toward a realistic pathway for poling-free organic devices to reach efficiencies competitive with inorganic crystals.

A critical requirement for practical deployment is the thermal and long-term stability of the organic film. While molecular orientation in spontaneously oriented organics is typically lost near the glass transition temperature (*21*) (*T_g_*), TPA-QCN has a *T_g_* of 110°C, comfortably exceeding typical telecom maximum operating temperatures (*51*) (e.g., 85°C). Furthermore, physical vapor deposition can yield ultrastable glasses (*52*) that retain orientation slightly above their conventional *T_g_*, a behavior observed in both TPA-QCN films and our newly improved compounds (*23*). By capping the active layer with a higher *T_g_* material like TCTA (153°C), this thermal robustness can be further enhanced (*31*). Measurements of a TCTA-capped film, similar to the one used in this study, confirm this effect, with the combined stack maintaining 90% of its SHG signal up to 129°C (fig. S3), providing a sufficient thermal budget for common nanofabrication processes and operating conditions. Alongside this thermal resilience, these films exhibit excellent ambient stability, showing no degradation over 200 days unencapsulated and displaying no signs of optical degradation under the pump powers used in this study or the high peak intensities (>30 GW/cm^2^) used in characterization studies (*19*, *23*).

Beyond efficiency, the primary advantage of this approach is its fabrication simplicity. By using a flow that relies entirely on low-temperature, back-end-of-line compatible processing, we show that this material class is viable for heterogeneous integration on arbitrary substrates. Although we used a strip-loaded architecture for this demonstration, the films are fully compatible with top-down nanofabrication. Fully etched channel waveguides (Supplementary Text), which offer tighter confinement and smaller bend radii can be realized using established dry etching techniques, such as reactive ion etching with oxygen-based chemistries (*53*).

This versatility opens a clear pathway for two distinct integration strategies: the fabrication of efficient monolithic organic photonic circuits (a natural evolution of the work shown here by adding an etching step) or the development of hybrid devices. This second path leverages scalable, heterogeneous integration with established centrosymmetric platforms (like silicon, silicon nitride, or alumina), where the organic film can be applied either as an evanescently coupled nonlinear cladding or to fill high-confinement slot waveguide geometries that maximize light-matter interaction.

## MATERIALS AND METHODS

### Waveguide fabrication

The fabrication process, illustrated in fig. S4, consists of two main stages: (1 to 2) thin-film deposition and (3 to 7) photolithographic patterning.

1) Substrate preparation: Silicon substrates (1 cm by 1 cm) with a 2-μm thermally grown wet oxide layer were used. The substrates were cleaned by sequential sonication in deionized (DI) water with soap, DI water, acetone, and lastly isopropanol. After cleaning, substrates were dried with a nitrogen gun and immediately treated with ultraviolet ozone for 15 min to ensure a clean, hydrophilic surface for deposition.

2) Thin-film deposition: The organic films were deposited in an Angstrom Engineering EvoVac resistive vacuum thermal evaporator. The TPA-QCN (Ambeed, >95% purity) was ground and thoroughly degassed in vacuum before deposition. The TCTA material (Luminescence Technologies, >99% purity) was used as received. Once a base pressure of $∼5\times 10^{-7}$ torr was reached, a 230-nm film of TPA-QCN was deposited, followed by a 15-nm film of TCTA. Deposition rates were 4 and 1 Å/s for TPA-QCN and TCTA, respectively. Film thicknesses were subsequently validated by variable angle spectroscopic ellipsometry.

3) Resist spin coating: Orthogonal Inc. OSCor 5020 organic-compatible resist was spin-coated onto the TCTA layer. The process consisted of a static dispense, a resist spreading step at 500 rpm for 5 s, and a main spin at 5000 rpm for 60 s.

4) Soft bake: The sample was soft-baked on a hotplate at 90° for 1 min.

5) Exposure: The resist was exposed using an i-line (365 nm) mask aligner in contact mode with a standard chromium photolithography mask. The total exposure dose was 45 mJ/cm^2^.

6) Postexposure bake: The sample was postexposure baked at 90° for 1 min.

7) Development: The pattern was developed by immersing the sample in a bath of Orthogonal Inc. Developer100 for 2 min with mild stirring, followed by a drying with a nitrogen gun.

### Waveguide characterization

A schematic of the experimental setup used for SHG characterization is shown in fig. S5. The pump source was a continuous-wave, C-band tunable laser (ID Photonics CoBrite DX1-SC) operating in the 1527.6- to 1568.6-nm range. The laser output was directed from a polarization-maintaining fiber through a collimator (Thorlabs F260APC-1550). The free-space beam’s polarization was set to TE (in-plane) using a half-wave plate (HWP1, Thorlabs WPH10ME-1550) and a linear polarizer (Pol1).

For alignment and to characterize transmission at the SH wavelength, a 780-nm laser diode (Thorlabs CP780S) was used. Its polarization was set to TM using a linear polarizer (Pol2) and coaligned with the C-band laser using a dichroic beam splitter (DM1, Thorlabs DMLP950).

A removable pellicle beam splitter (BS1) and a broadband illumination source were used to image the input facet onto a camera for initial alignment. During measurements, BS1 was removed. The combined beams were coupled into the cleaved facet of the waveguide using a long-working distance apochromatic objective [OBJ1, Mitutoyo MY50X-825, 50×, numerical aperture (NA) = 0.42]. The sample was mounted on a six-axis positioning stage for precise alignment. The on-chip input power (*P*_ω_) was determined by measuring the power before the input objective (OBJ1) and subtracting the coupling loss derived from the cut-back measurements.

The output from the waveguide facet was collected and collimated using a high-NA aspheric lens (L1, Thorlabs C330TMD-C, NA = 0.7). A second dichroic mirror (DM2, Thorlabs DMLP950) was used to separate the transmitted fundamental and generated SHG signals into two distinct paths.

1) Fundamental path (D2): The transmitted C-band light was filtered by a long-pass filter (LP2, Thorlabs FELH1400) to remove any residual alignment laser light. It was then passed through a linear polarizer (Pol4) set to TE to measure the transmitted pump power on a powermeter (D2, Thorlabs S122C).

2) SHG path (D1): The visible SHG signal was passed through a bandpass filter stack (SP1, Thorlabs FESH0850 and LP1, Thorlabs FELH0700) to isolate the SHG signal from the pump. A linear polarizer (Pol3) was used to confirm the TM polarization of the SHG. The power was then measured with a high-sensitivity silicon powermeter (D1, Thorlabs S130VC).

All power and wavelength sweeps were automated using a custom Python script that controlled the tunable laser and recorded the power meter readings from both D1 and D2.

### Waveguide mode and phase-matching simulations

Numerical simulations were performed using a commercial finite-element method solver, Comsol Multiphysics, to calculate the effective refractive indices (*n*_eff_), propagation losses, and mode profiles of the strip-loaded waveguide. The 2D cross section of the waveguide (as shown in Fig. 2B) was modeled in a computational domain with a width of 20 μm and a height of 15 μm. Perfectly matched layers were used as boundary conditions.

The TPA-QCN layer was modeled as a uniaxial anisotropic material, with its ordinary and extraordinary axes aligned with the in-plane and out-of-plane directions, respectively. The wavelength-dependent optical constants (*n* and *k*) for *n*_ord_ and *n*_ext_ were imported from experimental ellipsometry data (see Fig. 2A). The refractive indices for the SiO_2_, TCTA (fig. S2), and resist (fig. S2) layers were modeled as isotropic media using their respective measured ellipsometric data.

To generate the phase-mismatch map shown in fig. S1, a 2D parameter sweep was performed. The TPA-QCN layer height was varied from 180 to 250 nm, and the top resist strip width was varied from 1500 to 3000 nm.

For each (height and width) pair in the sweep, the eigenmode solver was run to find the effective indices of the fundamental TE_00_ mode at the pump wavelength (λ_p_) and the fundamental TM_00_ mode at the second-harmonic wavelength ($\lambda_{\text{SH}}=\lambda_{p}/2$). This was done for the C-band extents (1528 and 1568 nm) and the center wavelength (1550 nm).

The phase mismatch, $\Delta n=n_{\text{eff}}\left({\mathrm{TE}}_{00},\lambda_{p}\right)-n_{\text{eff}}\left({\text{TM}}_{00},\lambda_{\text{SH}}\right)$, was then calculated. The figure’s 2D color map plots this Δ*n* value for a pump wavelength of λ_p_ = 1550 nm. The contour lines show the perfect phase-matching condition (Δ*n* = 0) for the three simulated wavelengths.

## Supplementary Materials

### This PDF file includes:

Supplementary Text Figs. S1 to S9 References
