# Supplementary material for: Poling-free integrated second-order nonlinear optics with evaporated organic thin films
Source: Sci Adv. 2026 May 27;12(22):eaeg3170. doi: 10.1126/sciadv.aeg3170 (PMC13215164; doi:10.1126/sciadv.aeg3170)
Supplement: Supplementary file 1 — Supplementary Text Figs. S1 to S9 References [file sciadv.aeg3170_sm.pdf]

Science Advances
AAAS

Supplementary Materials for

Poling-free integrated second-order nonlinear optics with evaporated organic thin films

Pierre-Luc Thériault *et al.*

Corresponding author: Stéphane Kéna-Cohen, s.kena-cohen@polymtl.ca

Sci. Adv. **12**, eaeg3170 (2026)
DOI: 10.1126/sciadv.aeg3170

This PDF file includes:

Supplementary Text
Figs. S1 to S9
References

Supplementary Text

Phase-Matching in Channel Waveguides

While the main text demonstrates phase-matching in a strip-loaded geometry, the fundamental principle of leveraging giant birefringence to achieve fundamental-mode phase-matching is applicable to other waveguide geometries, such as fully-etched channel waveguides. To verify this, we performed finite-difference simulations for a rectangular channel waveguide (Figure S6a) consisting of a TPA-QCN core of varying height h and width w on a SiO_2 substrate with an air cladding.

Figure S6(b) shows the calculated effective index difference ($\Delta n = n_{eff}(TE_{00}, \lambda) - n_{eff}(TM_{00}, \lambda/2)$) for the fundamental TE_{00} and TM_{00} modes as a function of the channel width and height. Similar to the strip-loaded case, the large negative birefringence allows the $TM_{00}(2\omega)$ curve to cross the $TE_{00}(\omega)$ curve, creating a phase-matching condition ($\Delta n = 0$, solid black line) across a range of geometries.

Figure S6(c) and (d) show the simulated mode profiles for the phase-matched $TE_{00}(\omega)$ and $TM_{00}(2\omega)$ modes, respectively. Both modes are well-confined within the organic core, and their spatial overlap is high, confirming that efficient, poling-free SHG can also be realized in monolithic, etched organic waveguides.

Loss mechanisms

Our analysis attributes the measured propagation losses to three distinct mechanisms: substrate leakage, lateral leakage, and scattering.

- **Substrate leakage:** This is the dominant loss channel at the fundamental wavelength (λ_ω). Simulations (Fig. S7) indicate that the 2 μm SiO_2 buffer is insufficient to confine the TE_{00} mode, resulting in > 15 dB/cm of leakage into the silicon substrate. At the second harmonic ($\lambda_{2\omega}$), this leakage is significantly lower due to tighter optical confinement. Increasing the buffer thickness to > 6 μm suppresses this mechanism to negligible levels (< 0.1 dB/cm) for both wavelengths.

- **Lateral leakage:** A loss channel specific to the second harmonic is the coupling of the phase-matched TM_{00} mode into TE slab modes supported by the unpatterned film (*50*). Simulations

show this can exceed 10 dB/cm (Fig. S9). However, this coupling is resonant; by optimizing the waveguide width to avoid mode crossings, this leakage can be suppressed to < 0.1 dB/cm.

- **Scattering:** Scattering from lithographic sidewall roughness and polycrystalline grain boundaries affects both wavelengths. Our experiments on optimized substrates (Fig. S8) isolate this mechanism, revealing a current scattering limit of ≤ 5 dB/cm at 1550 nm. Eliminating the leakage pathways discussed above would reduce total losses to this scattering floor, improving the conversion efficiency by a factor of 2.3.

Efficiency with losses

In the main text, we report the length-normalized efficiency $\eta_L = 29$ %/W/cm^2, which is a figure of merit defined by the simple relation $P_{2\omega} = \eta_L P_\omega^2 L^2$. This definition does not account for propagation losses. Therefore, by using a longer(shorter) waveguide, a lower(higher) η_L would be obtained.

To find the true intrinsic efficiency, or to project the efficiency for a lower-loss device, we must model the SHG process in the presence of loss. We note that the following derivation assumes the pump is depleted primarily by propagation loss, and that depletion due to nonlinear conversion is negligible. We start with the differential equation for the generation of second-harmonic (SH) power, $P_{2\omega}$, at a position z in the waveguide, assuming perfect phase-matching ($\Delta k = 0$):

$$dP_{2\omega,\text{gen}}(z) = \eta_0 P_\omega^2(z) dz \tag{S1}$$

Here, $P_\omega(z)$ is the fundamental power at z, and η_0 is the intrinsic efficiency parameter in units of W^{-1}cm^{-1}.

The fundamental power $P_\omega(z)$ and the generated SH power $P_{2\omega}(z)$ both experience linear propagation loss, described by α_ω and $\alpha_{2\omega}$ (in units of cm^{-1}), respectively.

The fundamental power at any point z is:

$$P_\omega(z) = P_\omega(0)e^{-\alpha_\omega z} \tag{S2}$$

where $P_\omega(0)$ is the on-chip power at the start of the waveguide ($z = 0$).

The small amount of SH power $dP_{2\omega,\text{gen}}$ generated in a slice dz at position z is:

$$dP_{2\omega,\text{gen}}(z) = \eta_0 P_\omega^2(0)e^{-2\alpha_\omega z} dz \tag{S3}$$

This generated SH power must then propagate from z to the end of the waveguide at $z = L$, and it attenuates by a factor of $e^{-\alpha_{2\omega}(L-z)}$ during that propagation. The power $dP_{2\omega,\text{exit}}$ arriving at the end facet from that slice dz is:

$$dP_{2\omega,\text{exit}} = \left(\eta_0 P_\omega^2(0) e^{-2\alpha_\omega z} dz\right) \cdot e^{-\alpha_{2\omega}(L-z)} \tag{S4}$$

To find the total SH power at the exit $P_{2\omega}(L)$, we integrate this expression from $z = 0$ to L:

$$P_{2\omega}(L) = \int_0^L \eta_0 P_\omega(0)^2 e^{-2\alpha_\omega z} e^{-\alpha_{2\omega}(L-z)} dz \tag{S5}$$

Solving the integral gives:

$$P_{2\omega}(L) = \eta_0 P_\omega(0)^2 \left[\frac{e^{-2\alpha_\omega L} - e^{-\alpha_{2\omega} L}}{\alpha_{2\omega} - 2\alpha_\omega}\right] \tag{S6}$$

We now equate our measured, loss-affected power $P_{2\omega,\text{meas}} = \eta_L P_\omega(0)^2 L^2$ (from the main text) with the theoretical power from Equation S6:

$$\eta_L P_\omega(0)^2 L^2 = \eta_0 P_\omega(0)^2 \left[\frac{e^{-2\alpha_\omega L} - e^{-\alpha_{2\omega} L}}{\alpha_{2\omega} - 2\alpha_\omega}\right] \tag{S7}$$

We can now solve for the intrinsic efficiency, η_0 (in W^{-1}cm^{-1}):

$$\eta_0 = \eta_L L^2 \left[\frac{\alpha_{2\omega} - 2\alpha_\omega}{e^{-2\alpha_\omega L} - e^{-\alpha_{2\omega} L}}\right] \tag{S8}$$

This formula allows us to de-embed all propagation losses. To find the intrinsic length-normalized lossless efficiency ($\eta_{0,L}$ in W^{-1}cm^{-2}), we use the relationship $\eta_{0,L} = \eta_0 / L$:

$$\eta_{0,L} = \eta_L L \left[\frac{\alpha_{2\omega} - 2\alpha_\omega}{e^{-2\alpha_\omega L} - e^{-\alpha_{2\omega} L}}\right] \tag{S9}$$

This $\eta_{0,L}$ represents the length-normalized efficiency our device would have if there were no propagation losses.

Using this relation, we can then estimate the intrinsic length normalized efficiency of our device to be:

$$\begin{aligned}\eta_{0,L} &= (29) \cdot (0.17) \left[\frac{20/4.343 - 2(20/4.343)}{e^{-2(20/4.343)(0.17)} - e^{-(20/4.343)(0.17)}}\right] \\ &= 91\%\text{W}^{-1}\text{cm}^{-2}\end{aligned}$$

Note that propagation losses were converted from dB/cm to cm^{-1} using the formula $\alpha[\text{cm}^{-1}] = \alpha_p[\text{dB/cm}]/(10 \cdot \log_{10}(e)) \approx \alpha_p[\text{dB/cm}]/4.343$.

Finally, we can also estimate the efficiency we would obtain if the losses were lowered to 5 dB/cm by simply using a thicker oxide layer:

$$\eta_{L,5dB} = \frac{\eta_{0,L}}{L}\left[\frac{e^{-2\alpha_\omega L} - e^{-\alpha_{2\omega} L}}{\alpha_{2\omega} - 2\alpha_\omega}\right] = 68\ \%\text{W}^{-1}\text{cm}^{-2}$$

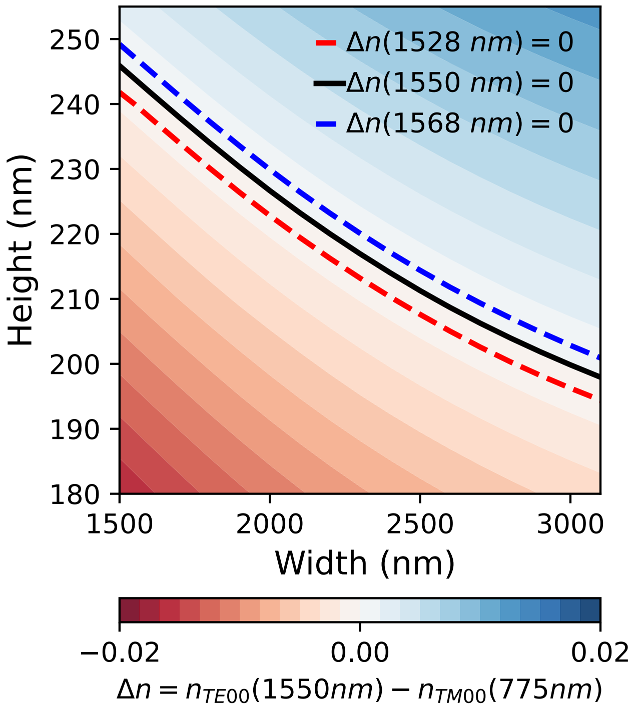

Figure S1: **Phase-mismatch map for the strip-loaded waveguide.** The 2D color map plots the effective index mismatch, $\Delta n = n_{eff}(TE_{00}, 1550\text{ nm}) - n_{eff}(TM_{00}, 775\text{ nm})$, as a function of TPA-QCN layer height and resist strip width. The contour lines show the perfect phase-matching condition ($\Delta n = 0$) for three different pump wavelengths: 1528 nm (red dashed), 1550 nm (black solid), and 1568 nm (blue dashed).

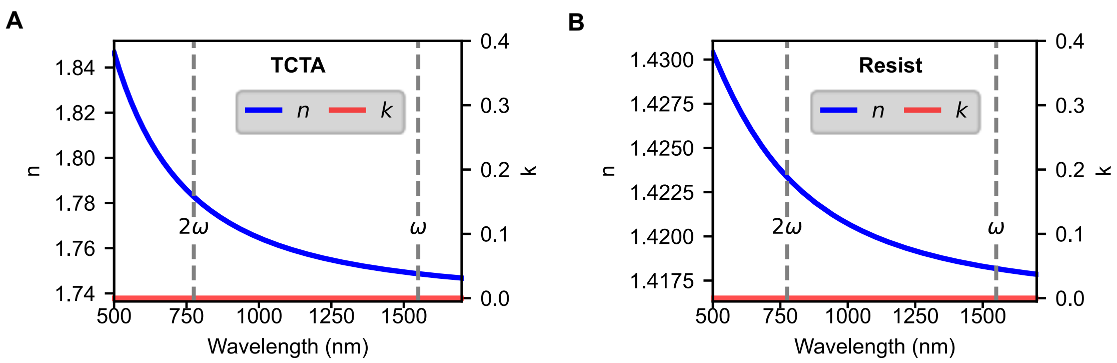

Figure S2: **Refractive Indices for the TCTA and Low-Index Resist** Real (n) and imaginary (k) refractive index for the **A** TCTA and **B** low-index resist. Both materials have a imaginary part that is zero over the plotted range.

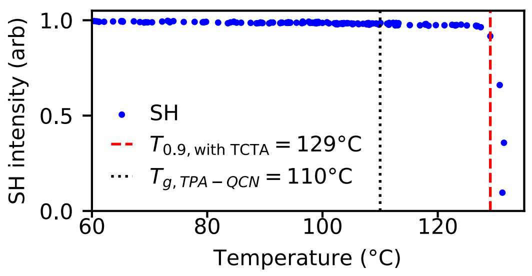

Figure S3: **Thermal stability of a TPA-QCN film capped with a thin TCTA layer.** Normalized second harmonic intensity as a function of temperature during heating (2.5°C/min) for a 150 nm film of TPA-QCN covered with 15 nm of TCTA. The black dotted line shows the glass transition temperature (T_g) of TPA-QCN, and the red dashed line indicates the temperature ($T_{0.9}$) at which the SHG signal has dropped to 90% of its initial value. Note that a slow (≈3%) degradation of the SHG signal occurs over time, which was absent during previous stability measurements at 1266 nm and 1550 nm (*23*) with >30GW/cm^2 pulses. This slow photoinduced degradation is likely due to two-photon-induced absorption (*54, 55*) of the strong 1030 nm fs pump pulses used for this particular characterization run.

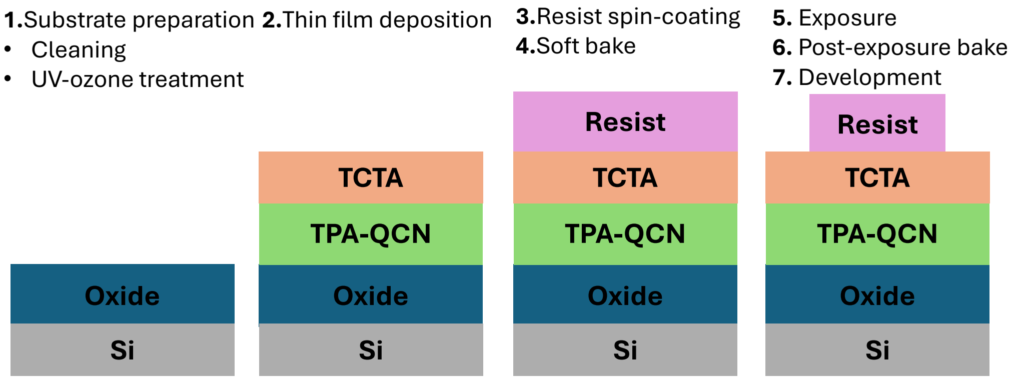

Figure S4: **Schematic of the strip-loaded waveguide fabrication process.** (1) Substrate preparation (cleaning and UV-ozone). (2) Resistive thermal evaporation of the TPA-QCN active layer and TCTA protective layer. (3) Spin-coating of the OSCor 5020 orthogonal resist. (4) Soft bake. (5) i-line UV exposure through a photomask. (6) Post-exposure (hard) bake. (7) Development of the resist to form the final strip-loaded waveguide.

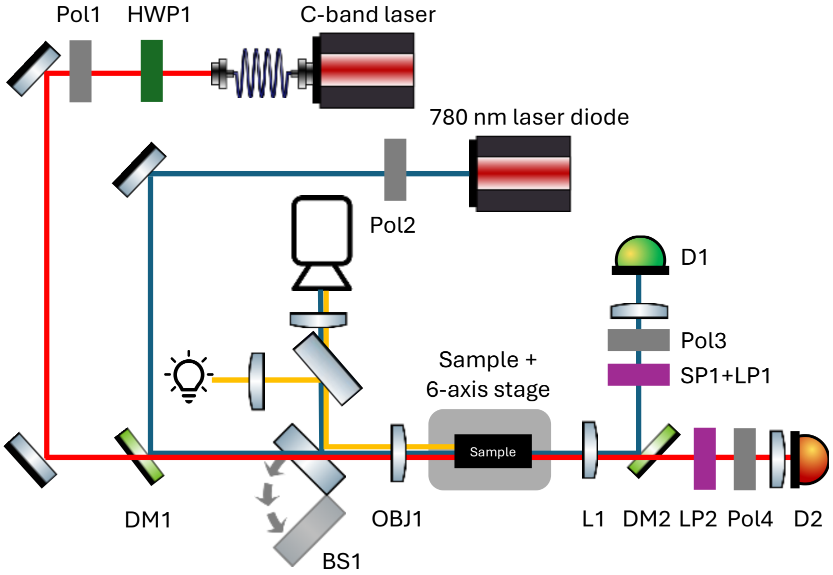

Figure S5: **Schematic of the optical characterization setup.** (HWP: Half-wave plate, Pol: Polarizer, DM: Dichroic mirror, BS: Beam splitter, OBJ: Objective, L: Lens, SP: Short-pass filter, LP: Long-pass filter, D: Detector/Powermeter).

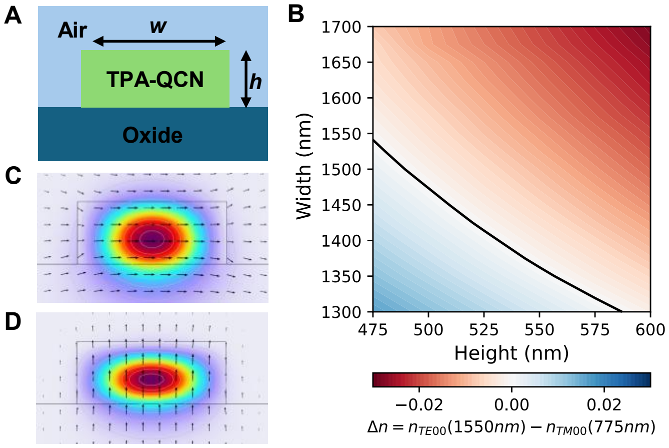

Figure S6: **Simulation of birefringent phase-matching in a TPA-QCN channel waveguide.** (a) Schematic of the channel waveguide geometry. (b) Phase-mismatch map (Δn) as a function of waveguide width and height for a pump wavelength of λ = 1550 nm. The solid black line indicates the perfect phase-matching condition ($\Delta n = 0$). (c, d) Mode profiles ($|E|^2$) for the phase-matched $TE_{00}(\omega)$ and $TM_{00}(2\omega)$ modes, respectively, showing strong confinement and overlap.

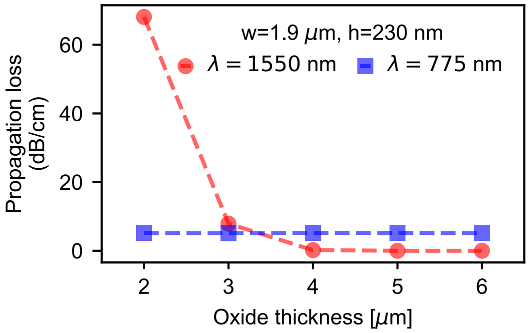

Figure S7: **Propagation loss simulations for different oxide thicknesses.** Simulation of the propagation losses versus oxide thickness length for a 1.9 μm waveguide with a 230 thick TPA-QCN layer. At 1550 nm, for the TE_{00} mode, substrate leakage is substantial for low oxide thickness and becomes negligible (< 0.001 dB/cm) for thicknesses exceeding 4 μm. At 775 nm, for the TM_{00} mode, substrate leakage is minimal and the main source of loss is lateral leakage.

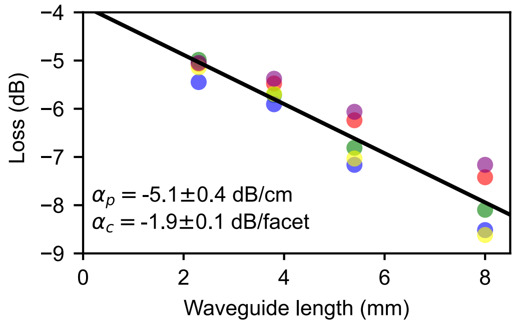

Figure S8: **Propagation loss characterization on a 6 μm oxide buffer.** Cut-back measurements of the total loss versus waveguide length performed at $\lambda = 1550$ nm. The waveguides were fabricated on a silicon substrate with a 6 μm thick thermal oxide layer to suppress substrate leakage. Different colors correspond to different waveguides. The linear fit yields a propagation loss of $\alpha_p = 5.1 \pm 0.4$ dB/cm and a coupling loss of $\alpha_c = 1.9 \pm 0.1$ dB/facet, confirming that losses are significantly reduced compared to the thin-buffer devices.

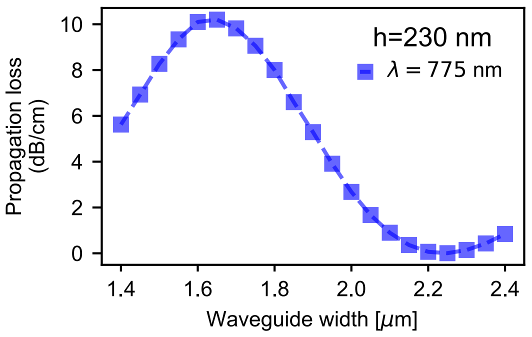

Figure S9: **Propagation loss simulation for different waveguide width.** Simulation of the propagation losses at 775 nm versus waveguide width (w) for a 230 thick TPA-QCN layer. For the TM_{00} mode, lateral leakage can exceed 10 dB/cm and be as low as 0.005 dB/cm depending on the width of the waveguide.

REFERENCES

1. S. Shekhar, W. Bogaerts, L. Chrostowski, J. E. Bowers, M. Hochberg, R. Soref, B. J. Shastri, Roadmapping the next generation of silicon photonics. *Nat. Commun.* **15**, 751 (2024).

2. D. A. Miller, Attojoule optoelectronics for low-energy information processing and communications. *J. Light. Technol.* **35**, 346–396 (2017).

3. Y. Shi, Y. Zhang, Y. Wan, Y. Yu, Y. Zhang, X. Hu, X. Xiao, H. Xu, L. Zhang, B. Pan, Silicon photonics for high-capacity data communications. *Photonics Res.* **10**, A106–A134 (2022).

4. Y. Shen, N. C. Harris, S. Skirlo, M. Prabhu, T. Baehr-Jones, M. Hochberg, X. Sun, S. Zhao, H. Larochelle, D. Englund, M. Soljačić, Deep learning with coherent nanophotonic circuits. *Nat. Photonics* **11**, 441–446 (2017).

5. B. J. Shastri, A. N. Tait, T. Ferreira de Lima, W. H. Pernice, H. Bhaskaran, C. D. Wright, P. R. Prucnal, Photonics for artificial intelligence and neuromorphic computing. *Nat. Photonics* **15**, 102–114 (2021).

6. A. W. Bruch, X. Liu, J. B. Surya, C.-L. Zou, H. X. Tang, On-chip $X^{(2)}$ microring optical parametric oscillator. *Optica* **6**, 1361–1366 (2019).

7. G. Moody, L. Chang, T. J. Steiner, J. E. Bowers, Chip-scale nonlinear photonics for quantum light generation. *AVS Quantum Sci.* **2**, 041702 (2020).

8. G. Sinatkas, T. Christopoulos, O. Tsilipakos, E. E. Kriezis, Electro-optic modulation in integrated photonics. *J. Appl. Phys.* **130**, 010901 (2021).

9. D. Zhu, L. Shao, M. Yu, R. Cheng, B. Desiatov, C. J. Xin, Y. Hu, J. Holzgrafe, S. Ghosh, A. Shams-Ansari, E. Puma, N. Sinclair, C. Reimer, M. Zhang, M. Lončar, Integrated photonics on thin-film lithium niobate. *Adv. Opt. Photonics* **13**, 242–352 (2021).

10. M. Li, L. Zhang, L.-M. Tong, D.-X. Dai, Hybrid silicon nonlinear photonics [Invited]. *Photonics Res.* **6**, B13 (2018).

11. P. Bettotti, Hybrid materials for integrated photonics. *Adv. Opt.* **2014**, 1–24 (2014).

12. Y. Tan, S. Niu, M. Billet, N. Singh, M. Niels, T. Vanackere, J. Van Kerrebrouck, G. Roelkens, B. Kuyken, D. Van Thourhout, Micro-transfer printed thin film lithium niobate (TFLN)-on-silicon ring modulator. *ACS Photonics* **11**, 1920–1927 (2024).

13. A. W. Elshaari, W. Pernice, K. Srinivasan, O. Benson, V. Zwiller, Hybrid integrated quantum photonic circuits. *Nat. Photonics* **14**, 285–298 (2020).

14. I. Biaggio, The appeal of small molecules for practical nonlinear optics. *Chem. A Eur. J.* **28**, e202103168 (2022).

15. T. W. Baehr-Jones, M. J. Hochberg, Polymer silicon hybrid systems: A platform for practical nonlinear optics. *J. Phys. Chem. C* **112**, 8085–8090 (2008).

16. L. R. Dalton, J. Leuthold, B. H. Robinson, C. Haffner, D. L. Elder, L. E. Johnson, S. R. Hammond, W. Heni, C. Hosessbacher, B. Baeuerle, E. De Leo, U. Koch, P. Habegger, Y. Fedoryshyn, D. Moor, P. Ma, Perspective: Nanophotonic electro-optics enabling THz bandwidths, exceptional modulation and energy efficiencies, and compact device footprints. *APL Materials* **11**, 050901 (2023).

17. I. Taghavi, M. Moridsadat, A. Tofini, S. Raza, N. A. F. Jaeger, L. Chrostowski, B. J. Shastri, S. Shekhar, Polymer modulators in silicon photonics: Review and projections. *Nanophotonics* **11**, 3855–3871 (2022).

18. J. Mao, H. Sato, A. Bannaron, J. Hong, G.-W. Lu, S. Yokoyama, Efficient silicon and side-cladding waveguide modulator with electro-optic polymer. *Opt. Express* **30**, 1885–1895 (2022).

19. P.-L. Thériault, A. Malinge, H. V. Humeniuk, D. Bourbonnais-Sureault, G. Juteau, R. Martel, D. F. Perepichka, S. Kéna-Cohen, Spontaneously-oriented evaporated organic semiconductor thin films for second-order nonlinear photonics. *ACS Photonics* **11**, 4297–4305 (2024).

20. A. Hofmann, M. Schmid, W. Brütting, The many facets of molecular orientation in organic optoelectronics. *Adv. Opt. Mater.* **9**, 2101004 (2021).

21. E. Pakhomenko, S. He, R. J. Holmes, Understanding and engineering spontaneous orientation polarization in organic light-emitting devices. *Chem. Phys. Rev.* **4**, 021308 (2023).

22. M. Tanaka, M. Auffray, H. Nakanotani, C. Adachi, Spontaneous formation of metastable orientation with well-organized permanent dipole moment in organic glassy films. *Nat. Mater.* **21**, 819–825 (2022).

23. P.-L. Thériault, H. V. Humeniuk, Z. He, G. Juteau, A. Malinge, D. F. Perepichka, S. Kéna-Cohen, Molecular engineering for enhanced second-order nonlinear response in spontaneously-oriented evaporated organic films. *Adv. Opt. Mater.* **14**, e02212 (2026).

24. R. W. Boyd, *Nonlinear Optics* (Academic Press, ed. 3, 2008).

25. F. Ye, Y. Yu, X. Xi, X. Sun, Second-harmonic generation in etchless lithium niobate nanophotonic waveguides with bound states in the continuum. *Laser Photonics Rev.* **16**, 2100429 (2022).

26. J. J. Chakkoria, R. A. Aoni, A. Dubey, G. Ren, T. G. Nguyen, A. Boes, S. K. Selvaraja, A. Mitchell, Efficient poling-free wavelength conversion in thin film lithium niobate harnessing bound states in the continuum. *Laser Photonics Rev.* **18**, 2301335 (2024).

27. L. Qu, W. Wu, W. Cai, M. Ren, J. Xu, Second harmonic generation in lithium niobate on insulator. *Laser Photonics Rev.* **19**, 2401928 (2025).

28. C. Li, R. Duan, B. Liang, G. Han, S. Wang, K. Ye, Y. Liu, Y. Yi, Y. Wang, Deep-red to near-infrared thermally activated delayed fluorescence in organic solid films and electroluminescent devices. *Angew. Chem. Int. Ed.* **56**, 11525–11529 (2017).

29. I. Shoji, T. Kondo, A. Kitamoto, M. Shirane, R. Ito, Absolute scale of second-order nonlinear-optical coefficients. *J. Opt. Soc. Am. B* **14**, 2268–2294 (1997).

30. C. Lu, Y. Zhang, J. Qiu, Y. Tang, T. Ding, S. Liu, Y. Zheng, X. Chen, Highly tunable birefringent phase-matched second-harmonic generation in an angle-cut lithium niobate-on-insulator ridge waveguide. *Opt. Lett.* **47**, 1081–1084 (2022).

31. J. Ràfols-Ribé, A. Vila-Costa, C. Rodríguez-Tinoco, A. F. Lopeandia, J. Rodríguez-Viejo, M. Gonzalez-Silveira, Kinetic arrest of front transformation to gain access to the bulk glass

transition in ultrathin films of vapour-deposited glasses. *Phys. Chem. Chem. Phys.* **20**, 29989–29995 (2018).

32. A. Rao, K. Abdelsalam, T. Sjaardema, A. Honardoost, G. F. Camacho-Gonzalez, S. Fathpour, Actively-monitored periodic-poling in thin-film lithium niobate photonic waveguides with ultrahigh nonlinear conversion efficiency of $4600\%/W/cm^2$. *Opt. Express* **27**, 25920–25930 (2019).

33. J. Hill, P. Dunn, G. Davies, S. Oliver, P. Pantelis, J. Rush, Efficient frequency-doubling in a poled PVDF copolymer guest/host composite. *Electron. Lett.* **23**, 700–701 (1987).

34. G. Hewig, K. Jain, Frequency doubling in an organic waveguide. *Opt. Commun.* **47**, 347–350 (1983).

35. C. Flueraru, S. Schrader, B. Dietzel, H. Motschmann, Phase-matched second harmonic generation and cascaded nonlinearity in a Langmuir-Blodgett inverted waveguide of 2-docosylamino-5-nitropyridine. *J. Appl. Phys.* **90**, 5469–5477 (2001).

36. H. Yamamoto, S. Funato, T. Sugiyama, J. Jung, T. Kinoshita, K. Sasaki, Organic crystal isopropyl-4-acetylphenylurea and waveguide second-harmonic generation. *J. Opt. Soc. Am. B* **14**, 1099–1108 (1997).

37. J. J. Ju, S. K. Park, S. Park, J. Kim, M.-s. Kim, M.-H. Lee, J. Y. Do, Wavelength conversion in nonlinear optical polymer waveguides. *Appl. Phys. Lett.* **88**, 241106 (2006).

38. L. Alloatti, D. Korn, C. Weimann, C. Koos, W. Freude, J. Leuthold, Second-order nonlinear silicon-organic hybrid waveguides. *Opt. Express* **20**, 20506–20515 (2012).

39. M. Jäger, G. Stegeman, W. Brinker, S. Yilmaz, S. Bauer, W. Horsthuis, G. Möhlmann, Comparison of quasi-phase-matching geometries for second-harmonic generation in poled polymer channel waveguides At 1.5 Mu M. *Appl. Phys. Lett.* **68**, 1183–1185 (1996).

40. M. Jäger, G. Stegeman, S. Yilmaz, W. Wirges, W. Brinker, S. Bauer-Gogonea, S. Bauer, M. Ahlheim, M. Stähelin, B. Zysset, F. Lehr, M. Diemeer, M. C. Flipse, Poling and

characterization of polymer waveguides for modal dispersion phase-matched second-harmonic generation. *J. Opt. Soc. Am. B* **15**, 781–788 (1998).

41. M. L. Jager, "Efficient second-harmonic generation in poled polymer waveguides using copropagating geometries," thesis, University of Central Florida (1997).

42. S. Fukuda, T. Gotoh, Vapor phase grown second-order nonlinear optical organic crystal film waveguides. *Rev. Laser Eng.* **21**, 1134–1141 (1993).

43. T. Suhara, T. Morimoto, H. Nishihara, Optical second-harmonic generation by quasi-phase matching in channel waveguide structure using organic molecular crystal. *IEEE Photon. Technol. Lett.* **5**, 934–937 (1993).

44. O. Sugihara, K. Sasaki, Phase-matched second-harmonic generation in a 2-methyl-4-nitroaniline single-crystal waveguide: Combined structure of grating couplers and four-layer waveguide. *J. Opt. Soc. Am. B* **9**, 104–107 (1992).

45. H. Figi, M. Jazbinšek, C. Hunziker, M. Koechlin, P. Günter, Electro-optic single-crystalline organic waveguides and nanowires grown from the melt. *Opt. Express* **16**, 11310–11327 (2008).

46. H. Figi, M. Jazbinšek, C. Hunziker, M. Koechlin, P. Günter, Electro-optic tuning and modulation of single-crystalline organic microring resonators. *J. Opt. Soc. Am. B* **26**, 1103–1110 (2009).

47. W. Geis, R. Sinta, W. Mowers, S. Deneault, M. Marchant, K. Krohn, S. Spector, D. Calawa, T. Lyszczarz, Fabrication of crystalline organic waveguides with an exceptionally large electro-optic coefficient. *Appl. Phys. Lett.* **84**, 3729–3731 (2004).

48. T. Kaino, B. Cai, K. Takayama, Fabrication of DAST channel optical waveguides. *Adv. Funct. Mater.* **12**, 599–603 (2002).

49. N. Krainova, H. M. Johnson, R. Lampande, S. Gao, N. Marom, B. P. Rand, N. C. Giebink, Vacuum deposition of $X^{(2)}$ nonlinear organic single crystal films on silicon. *Appl. Phys. Lett.* **125**, 031104 (2024).

50. T. G. Nguyen, A. Boes, A. Mitchell, Lateral leakage in silicon photonics: Theory, applications, and future directions. *IEEE J. Sel. Top. Quantum Electtron.* **26**, 1–13 (2020).

51. C. Kieninger, Y. Kutuvantavida, H. Miura, J. N. Kemal, H. Zwickel, F. Qiu, M. Lauermann, W. Freude, S. Randel, S. Yokoyama, C. Koos, Demonstration of long-term thermally stable silicon-organic hybrid modulators at 85 °C. *Opt. Express* **26**, 27955–27964 (2018).

52. S. S. Dalal, D. M. Walters, I. Lyubimov, J. J. de Pablo, M. D. Ediger, Tunable molecular orientation and elevated thermal stability of vapor-deposited organic semiconductors. *Proc. Natl. Acad. Sci. U.S.A.* **112**, 4227–4232 (2015).

53. M. Höppner, D. Kneppe, H. Kleemann, K. Leo, Precise patterning of organic semiconductors by reactive ion etching. *Org. Electron.* **76**, 105357 (2020).

54. M. Cha, W. E. Torruellas, G. I. Stegeman, W. H. G. Horsthuis, G. R. Möhlmann, J. Meth, Two photon absorption of di-alkyl-amino-nitro-stilbene side chain polymer. *Appl. Phys. Lett.* **65**, 2648–2650 (1994).

55. Y. Tominari, T. Yamada, T. Kaji, C. Yamada, A. Otomo, Photostability of organic electro-optic polymer under practical high intensity continuous-wave 1550 nm laser irradiation. *Jpn. J. Appl. Phys.* **60**, 101002 (2021).
